# 

**Published:** 1904-08

**Authors:** 


VOL. LX.

WHOLE NUMBER,

DCLXXXXIII

AUGUST, 1904.

New Series.

VOL. XLIV

No. 1

### BUFFALO MEDICAL JOURNAL

Established 1845 by AUSTIN FLINT, M. D.

LIBRARY
SURGEON GENERAL'S OFFICE
AUG. -1. 1904

LIBRARY
INDEXED
S. G. O.

EDITOR:

WILLIAM WARREN POTTER, M. D.

ASSISTANT EDITORS:
WILLIAM C. KRAUSS, M. D. NELSON W. WILSON, M. D.

ASSOCIATE EDITORS:

JAMES WRIGHT PUTNAM, M. D. ERNEST WENDE, M. D.

JOHN PARMENTER, M. D. JOHN A. MILLER, PH. D.

HARVEY R. GAYLORD, M. D. MAUD J. FRYE, M. D.

For terms and other information relating to the Journal, see page vi.

